# Goal pursuit increases more after dietary success than after dietary failure: examining conflicting theories of self-regulation using ecological momentary assessment

**DOI:** 10.1186/s12966-024-01566-x

**Published:** 2024-02-26

**Authors:** Hannah van Alebeek, Christopher M. Jones, Julia Reichenberger, Björn Pannicke, Benjamin Schüz, Jens Blechert

**Affiliations:** 1 Department of Psychology, Centre for Cognitive Neuroscience, Paris-Lodron-University of Salzburg, Hellbrunner Str. 34, 5020 Salzburg, Austria; 2 Medical Faculty Mannheim, Heidelberg University, Heidelberg, Germany; 3 Institute for Public Health and Nursing Research, University of Bremen, Bremen, Germany

**Keywords:** Self-regulation, Ecological momentary assessment, Cybernetic model, Social cognitive theory, Intentions, Regulatory effort, Self-monitoring, Dietary lapse, Food intake

## Abstract

**Background:**

Maintaining a healthy body weight and reaching long-term dietary goals requires ongoing self-monitoring and behavioral adjustments. How individuals respond to successes and failures is described in models of self-regulation: while cybernetic models propose that failures lead to increased self-regulatory efforts and successes permit a reduction of such efforts, motivational models (e.g., social-cognitive theory) make opposite predictions. Here, we tested these conflicting models in an ecological momentary assessment (EMA) context and explored whether effort adjustments are related to inter-individual differences in perceived self-regulatory success in dieting (i.e., weight management).

**Methods:**

Using linear mixed effects models, we tested in 174 diet-interested individuals whether current day dietary success or failure (e.g., on Monday) was followed by self-regulatory effort adjustment for the next day (e.g., on Tuesday) across 14 days. Success vs. failure was operationalized with two EMA items: first, whether food intake was higher vs. lower than usual and second, whether food intake was perceived as more vs. less goal-congruent than usual. Trait-level perceived self-regulatory success in dieting was measured on a questionnaire.

**Results:**

Intended self-regulatory effort increased more strongly after days with dietary success (i.e., eating less than usual / rating intake as goal-congruent) than after days with dietary failure (i.e., eating more than usual / rating intake as goal-incongruent), especially in those individuals with lower scores on perceived self-regulatory success in dieting.

**Conclusions:**

Findings support mechanisms proposed by social-cognitive theory, especially in unsuccessful dieters. Thus, future dietary interventions could focus on preventing the decrease in self-regulatory effort after instances of dietary failures and thereby mitigate the potential risk that a single dietary failure initiates a downward spiral into unhealthy eating.

**Supplementary Information:**

The online version contains supplementary material available at 10.1186/s12966-024-01566-x.

## Introduction

Understanding the processes of how behavior can be changed to achieve health-related goals is crucial to improve interventions targeting unhealthy lifestyles and related diseases. For example, in the case of obesity and overweight, improving dietary patterns could significantly reduce the risk for a range of sometimes even fatal diseases such as diabetes, or musculoskeletal and cardiovascular diseases [1]. According to theories of self-regulation, key processes in achieving goal-oriented behavior change involve monitoring one’s progress and adapting behavioral effort accordingly when striving towards a goal (e.g., [2–5]). Here, self-monitoring can be seen as an ongoing process which repeatedly takes place over time [6]. For example, someone intending to lose five kilos of body weight would repeatedly monitor whether dietary changes lead to the desired weight loss and make suitable adjustments (i.e., invest more effort) if not on track. Yet, how this iterative process of self-regulatory effort, goal-directed behavior and subsequent feedback plays out over time remains unclear. For example, what happens when a person trying to lose five kilos finds their progress stagnant? Will they give up or instead increase their effort for low calorie intake? Surprisingly, existing self-regulation theories make conflicting predictions on how behavioral effort is adjusted to perceived dietary success vs. failure.

**Fig. 1:**
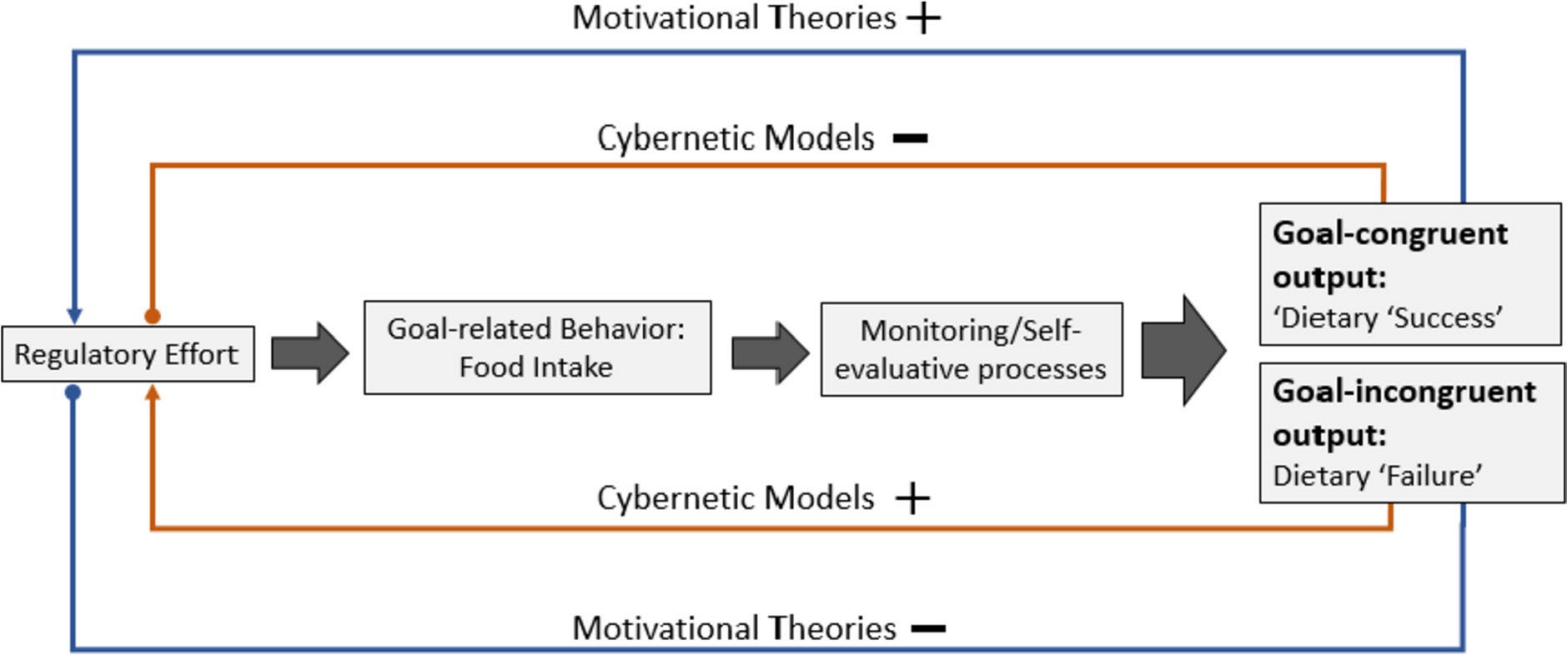
Hypothesized relationships between goal-congruent and goal-incongruent output on subsequent self-regulatory efforts

*Cybernetic models* of self-regulation (e.g., [3]) are perhaps the most influential ‘classic’ models of self-regulation and are based on simple feedback loops with four key elements: Individuals compare their 1) current state of goal attainment with a 2) set standard/goals within a 3) discrepancy monitoring system that exports to a 4) implementing system to reduce potential discrepancies between goal and current state [7]. The four elements constitute an iterative feedback loop, as implemented changes are continuously monitored for their effects on goal achievement. Goal-*in*congruent behavior should trigger negative affect and the need to reduce the discrepancy between the current state and the goal, thereby increasing self-regulatory effort and goal pursuit. Identification of goal-*con*gruent behavior, by contrast, should trigger positive affect and provide the individual with a sense of (partial) goal attainment, thereby reducing self-regulatory effort (so-called coasting; [3]). Thus, self-regulatory effort is used to calibrate behavior towards the goal (termed ‘calibrating hypothesis’ herein; see ‘Cybernetic Models’ in Fig. 1).

*Motivational theories*, e.g. Social Cognitive Theory (SCT; [2]), or the attributional theory of motivation [8] in contrast assume that goal-incongruent behavior – especially if attributed to the self – undermines an individual's self-efficacy and positive outcome expectancies, which in turn leads to *lower* self-regulatory effort and goal striving whereas goal-congruent behavior – again, especially if attributed to the self – induces stronger self-efficacy through mastery-experience which in turn leads to positive outcome expectancies, positive affect, and stronger subsequent self-regulatory efforts such as stronger intentions [9, 10]. Thus, instead of calibrating self-regulatory effort to goals, motivational theories assume that monitoring of goal-congruency *can* initiate self-reinforcing down- or upward spirals that paradoxically lead to significant deviations from the initially set behavioral standard (termed here as the ‘self-reinforcing hypothesis’; ‘Motivational Theories’ in Fig. 1).

Self-regulation is particularly relevant for successful dietary management. Many people struggle in following diet-related goals because external (e.g., food smells, advertisement) and internal cues (e.g., negative emotion, stress) can trigger urges to eat in the absence of physiological needs [11, 12]. This susceptibility to eating cues, motivated extensive laboratory research on how inhibitory control – as one instance of self-regulatory effort – helps to resist the urge of overeating [13, 14]. What remains unclear, however, is whether – and how – self-regulation of eating is maintained over time [15], that is, how past instances of dietary ‘failure’ or ‘success’ (i.e., goal-congruent and goal-incongruent behavior) affect future goal striving across days.

In line with the cybernetic ‘calibrating hypothesis’ of cybernetic models that monitoring of goal-*in*congruent behavior serves as a signal for *more* effort towards the goal, intervention studies showed that monitoring of food intake and providing feedback on instances of dietary ‘failure’ – also called dietary lapse – proved beneficial for long-term weight loss and healthy food intake [16]. Furthermore, individuals who experience negative affect after a dietary lapse (i.e., self-criticism) are less likely to experience another dietary lapse on the same day [17]. This indicates that negative affective feedback after goal-incongruent eating may signal a necessity for increased regulatory effort. Regarding goal-*con*gruent behavior, an experimental study found lower intentions to lose weight after reflecting on past dietary success (i.e., goal-congruent eating; Study 1; [18]).

However, there is also evidence for the ‘self-reinforcing hypothesis’ derived from motivational theories. Higher self-efficacy is related to (intended) healthier eating [19, 20] and interventions that increase self-efficacy have been successful in improving dietary patterns and weight loss [21]. This supports the idea that processes that increase self-efficacy, such as achieving one´s dietary goals, are beneficial to successful regulation of subsequent food intake. Yet, it has not yet been specifically tested whether past instances of goal-*con*gruent eating – or dietary ‘success’ – indeed increase self-efficacy and self-regulatory effort over time. There is, however, some evidence that goal-*in*congruent behavior – or dietary ‘failure’ – may lead to *lower* subsequent self-regulatory effort and goal striving: Participants who gave into food temptations reported a subsequent decrease in self-regulatory abilities (e.g., self-efficacy, perceived behavioral control, willpower; [22–24]), and individuals with overweight who felt guilty or less self-compassionate after experiencing a dietary ‘failure’ decreased their intentions to follow their dietary plans [25]. With intentions being a key predictor of behavior [4, 26], these results are in line with another study showing that women who felt less positive about themselves after overeating were more prone to subsequent overeating [27], conceptually conflicting with [17]. Together, these studies conflict with cybernetic models and suggest that perceived dietary ‘failure’ might induce counter-regulatory and disinhibited eating through negative affect or negative self-evaluative cognitions (as with the abstinence violation effect; [28]).

Heterogeneous evidence and conflicting theoretical predictions limit our understanding of the dynamic processes relevant for long-term dietary success and weight loss. To better capture how past eating behavior may feed back to subsequent self-regulatory effort, we analyzed data from two prior ecological momentary assessment (EMA) studies.

Based on cybernetic and motivational theories, we tested two contradictory hypotheses:

1. Calibrating hypothesis: Dietary ‘failure’ (i.e., not reaching a behavioral standard/goal) leads to subsequent *increases* in intended dietary effort whereas dietary ‘success’ (i.e., meeting or outperforming a behavioral standard/goal) leads to subsequent *decreases* in intended dietary effort.
2. Self-reinforcing hypothesis: Dietary ‘failure’ leads to subsequent *decreases* in intended dietary effort whereas dietary ‘success’ leads to subsequent *increases* in intended dietary effort.

Dietary ‘success’ or ‘failure’ can be operationalized in several ways. Here, we used two measures, first, participants´ self-evaluations of how much their intake was in line with their dietary goals (‘explicit’ monitoring / appraisals), and second, participants’ self-reported amount of food intake on days with healthy eating intentions (‘implicit’ monitoring / appraisals). Such an ‘explicit’ monitoring / appraisal model allowed us to test whether changes in self-regulatory efforts can be attributed to perceived self-evaluative processes as described in cybernetic and motivational theories, and the ‘implicit’ monitoring / appraisal model whether actual dietary behavior, namely food intake, is relevant for changes in self-regulatory efforts. Furthermore, we explored whether food type (fruit/vegetables vs. sweets/snacks) moderated how food amount relates to subsequent effort adjustments (i.e., eating a large amount of fruits and vegetables may count as a dietary ‘success’ whereas eating a large amount of sweets and snacks may count as a dietary ‘failure’), and whether trait differences in self-regulation for dieting (i.e., weight management) moderates these moment-to-moment effort adjustments derived from the EMA data.

## Methods

To test these hypotheses, we re-analyzed data from two previously published EMA studies on everyday food consumption [29–32]. Participants in these studies expressed the desire to lose or maintain weight which ensured that they were sufficiently motivated to trigger self-regulatory processes. Both studies were combined as they shared recruitment and assessment procedures as well as most items used. Analyses separated by data set can be found in Additional file 1: Appendix A. Study 1 was a randomized controlled trial (preregistered at the German register of clinical studies: DRKS, DRKS0001749) in which the intervention group differed from the control group by receiving daily eating tips in addition to the EMA items included in both groups. Study 2 was purely observational. Because we were interested in spontaneously occurring dietary behavior, we included all participants from study 2, but only those who did not receive any eating tips were included from study 1.

### Participants

Participants of both studies were recruited via posts on social media platforms and via e-mail study announcements at several universities in Austria and Germany. To over-sample diet-motivated individuals, potential participants were only allowed to enroll in the study when they agreed to at least one of the following two sentences: 1) 'Do you currently pay attention to your nutrition in order to maintain or reduce your body weight?' and 2) 'Do you currently cut down on your food intake in order to maintain or reduce your body weight?'. Further, participants in study 1 were required to own a smartphone running Android to be able to install and run the customized EMA app. The first dataset includes 91 participants (12% identified as male) and the second one 83 participants (13% identified as male). Further characterization of the samples can be found in the results section.

### Procedure

Data for both studies were collected at the University of Salzburg following an identical protocol. After participants had received written and oral information on the study protocol and signed an informed consent form according to the guidelines of the ethical committee of the University of Salzburg, which also approved the studies procedures, they completed several trait measures via the online platform LimeSurvey. Subsequently, the EMA period started (described below). In both studies, participants received individualized feedback or course credit for participation. To motivate compliance, participants within the top 20% compliance rate additionally received 25 Euro.

### Measures

#### Study 1: EMA

Participants completed 14 consecutive days of EMA with 6 time-contingent EMA-questionnaires per day (at 9:00, 11:30, 14:00, 16:30, 19:00, and 21:30). App push notifications prompted participants to indicate the amount of food intake since the last prompt (*Food intake*: ‘How much did you eat?’), how much the intake was in line with their dietary goals (*goal-congruent eating*: ‘How much did this eating episode correspond to your eating goal?’). In addition to the report of overall amount of food intake, participants classified their amount of intake, using categories including sweets, salty snacks, nuts, fruits, and vegetables. Evening prompts at 21:30 required reports of *intended effort* to eat in line with dietary goals during the next day (‘How much do you want to actively manage your food-related behavior towards your weight goal tomorrow?’). All items were answered on a horizontal slider ranging from 0 (= ‘very little’/ ‘not at all’) to 100 (= ‘very much’/ ‘very’). EMA-items not included in the current analyses such as craving, hunger or affect are described elsewhere [29, 30].

#### Study 2: EMA

EMA in study 2 was of similar length (14 days), but with only 4 time-contingent EMA-questionnaires per day (9:00, 13:00, 17:00, and 21:00). While the general item pool differed between both studies (e.g., the second study also included a range of items regarding participants´ physical activity), the items included in the current analyses had equivalent wording. As in study 1, the item regarding future self-regulatory effort was shown in the evenings, whereas all other items were prompted during all four daily assessments. EMA-items not included in the study are described elsewhere [31, 32].

#### Perceived self-regulatory success in dieting scale (PSRS)

To assess inter-individual differences in self-regulation for dieting (i.e., weight management), we used the PSRS [33]. Participants rated how successful they typically are at losing weight, controlling their weight, and maintaining their shape on three items ranging from 1 (‘not at all’) to 7 (‘very much’). Internal consistency was acceptable across datasets (α_study 1_ = 0.7, ω_study 1_ = 0.73, α_study 2_ = 0.72, ω_study 2_ = 0.75).

#### Additional questions to characterize the sample

Apart from other questionnaires that are not pertinent to the current study, we obtained the following measures to characterize our sample: The Dutch Eating Behavior Questionnaire assessed restrained, emotional and external eating [34], the Salzburg Stress Eating Scale assessed stress-related eating [35], and the Trait Food Craving Questionnaire assessed food cravings [36]. We additionally obtained demographic information (i.e., age, years of education, height, and weight), as well as responses to two items assessing the emphasis participants place on being slim (‘How important is it to you to be slim?’), and regulating their weight (‘How important is it to you to track your weight?’). 

### Data analyses

**Fig. 2:**
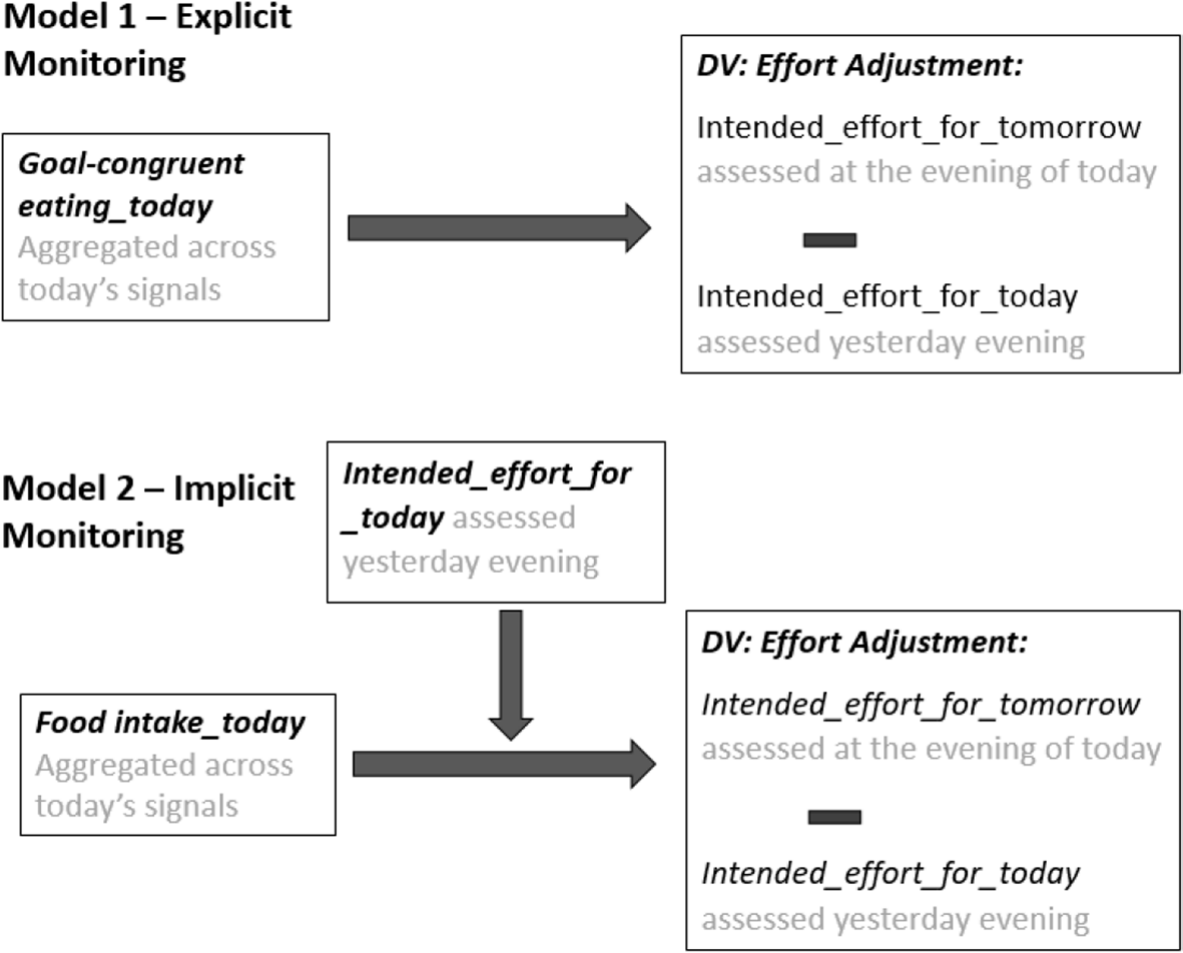
Illustration of statistical models

**Fig. 3:**
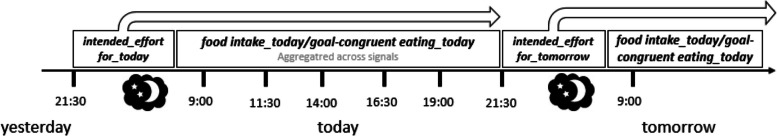
Timeline of relevant EMA variables

Data and analyses scripts are available online (https://osf.io/p2kq5/). Due to the hierarchical structure of the data with measurements nested within participants, we used linear mixed effect models with random intercepts and slopes for main and interaction effects, treating days (level-1 units) as nested within participants (level-2 units). To decompose within- and between-participant effects for all level-1 predictors, we included a) person-means (between subjects, ‘bs’) and b) each level-1 predictor centered on this mean (within subjects, ‘ws’; [37]). Our independent variables food intake (f*ood intake_today*) and goal-congruency of eating (*goal-congruent eating_today*) were computed by averaging across all signals of that day. As our dependent variable (Fig. 2), we took tonight’s rating on the intended effort for tomorrow (*intended_effort_for_tomorrow*) and subtracted yesterday’s rating on the intended effort for today (*intended_effort_for_today*). A positive value of this difference score (*effort_adjustment*) represents an increase in the intended self-regulatory effort from one day (i.e., today) to the next (i.e., tomorrow), while a negative value indicates a decrease. Figure 3 illustrates the timeline of our variables.

Model 1 (Fig. 2: explicit monitoring / appraisal; equation 1) examined whether adjustments in self-regulatory effort resulted from participants´ evaluation of their eating as goal-congruent or incongruent (*goal-congruent eating_today*). Model 2 (Fig. 2: implicit monitoring / appraisal) examined whether adjustments in self-regulatory effort resulted from the amount of food intake (*food intake_today*). As intake can only serve as an indicator for dietary ‘success’ or ‘failure’ on days on which participants actually tried to eat according to their goal (i.e., when a behavioral standard was set), we included an interaction between *food intake_today* and *intended_effort_for_today* (equation 2) in our prediction of effort adjustments. Interaction terms were further decomposed with Johnson-Neyman plots [38] to test at which level of the moderator (i.e., intended effort for the day), the relationship between the predictor (i.e., food intake) and the dependent variable (i.e., effort adjustments) was significant. To produce unbiased Type 1 error rates, *p*-values for fixed effects were based on *t*-tests with Satterthwaite corrections [39]. Compliance was above 80% in both studies (Study 1: *M* = 81%, *SD* = 0.19, Min = 14%, Max = 100%; Study 2: *M* = 85%, *SD* = 0.15, Min = 36%, Max = 100%). Reasons for missed prompts are however unknown. As prompts may not be missing completely at random, we analyzed whether our results were sensitive to different cut-offs for compliance (see Additional file 1: Appendix B). This was not the case. Specifically, findings remained unaffected by successively excluding participants with low compliance until the sample size decreased by more than 25%.

1. *Effort_adjustment* ~ *Goal-congruent eating_today (ws)* + *Intended_effort_for_today (ws)* + *Goal-congruent eating_today (bs)* + *Intended_effort_for_today (bs)* + *Day* + *Dataset* + *(Goal-congruent eating_today (ws)* + *Intended_effort_for_today (ws)* + *Day | Subject)*
2. *Effort_adjustment* ~ *Food Intake_today (ws)* × *Intended_effort_for_today (ws)* + *Food Intake_today (bs)* + *Intended_effort_for_today (bs)* + *Day* + *Dataset* + *(Food Intake_today (ws)* × *Intended_effort_for_today (ws)* + *Day | Subject)*.

## Results

### Sample characterization

The two samples did not differ with regard to their emotional, restrained, external and stress eating styles, and had similar trait-level food craving, perceived success in dieting, years of education and age (Welch´s t-test: Table 1). Despite both samples having Body Mass Indices classified as ‘normal’ by the WHO, participants of the second study had a significantly lower Body Mass Index.

**Table 1:** Comparison of the sample’s characteristics across the two studies

	Study			
	*Dataset 1*	*Dataset 2*		
*Questionnaires*	*M (SD)*	*M (SD)*	*t (df)*	*p*
Age	23.18 (3.64)	22.67 (4.14)	-.86 (164.35)	.390
Body Mass Index	23.11 (3.61)	21.92 (2.72)	-2.46 (167.79)	**.015**
Years of education	15.38 (2.47)	15.24 (2.76)	.34 (165.67)	.736
Restrained Eating	2.94 (0.68)	2.84 (0.78)	-.84 (163.48)	.400
External Eating	3.17 (0.59)	3.30 (0.66)	1.34 (165.48)	.182
Emotional Eating	2.62 (0.86)	2.70 (0.80)	.66 (172.83)	.513
Stress Eating	3.09 (0.75)	3.11 (0.68)	.14 (172.99)	.892
Food craving	2.87 (0.84)	2.92 (0.96)	.34 (164.47)	.737
Perceived self-regulatory success in dieting	4.06 (0.75)	4.12 (0.67)	.55 (172.95)	.585

Regarding inclusion criteria (being motivated to adhere to a diet), most participants reported reducing food intake (94.83%), with around half of them also regulating their nutritional intake (52.87%). Only 5.17% solely focused on regulating their nutrition. We additionally examined participants’ tendency to restrain their eating behavior compared to a normative sample within the same weight range using the Dutch Eating Behavior Questionnaire. Participants reported higher levels of restrained eating than the normative sample: *M* = 2.98 (*SD* = 0.73) versus *M* = 2.08 (*SD* = 0.91), *t* (1283) = 12.43, *p* < 0.001. Furthermore, participants placed substantial importance on being slim and regulating weight, as responses significantly surpassed the scales midpoint of 3.5 (being slim: *M* = 5.80, *SD* = 1.21, *t* (174) = 28.10, *p* < 0.001; regulating weight: *M* = 5.53, *SD* = 1.14, *t* (174) = 25.81, *p* < 0.001).

### Dietary ‘failure’ vs. ‘success’ predicts adjustments in self-regulatory effort

**Fig. 4:**
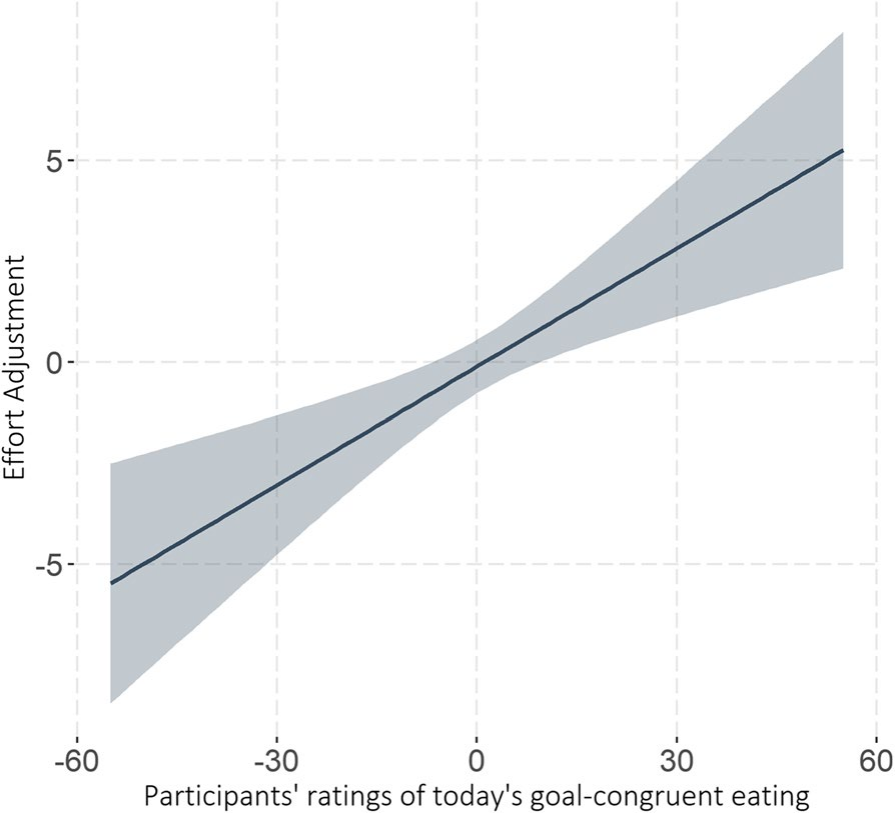
Self-regulatory effort increased more after goal-congruent (i.e., dietary ‘success’) than after goal-incongruent eating (i.e., dietary ‘failure’)

The model testing whether today’s goal-congruent or goal-incongruent eating influenced effort adjustments for tomorrow (Model 1: explicit monitoring / appraisal) yielded a main effect of *goal-congruent eating_today* (*b* = 0.10, β = 0.08, *t* (142.6) = 3.66, *p* < 0.001). This indicates that self-regulatory effort *in*creased more strongly after days with goal-congruent evaluations (i.e., dietary ‘success’) than after days with goal-incongruent evaluations (i.e., dietary ‘failure’), as seen in Fig. 4.

**Fig. 5:**
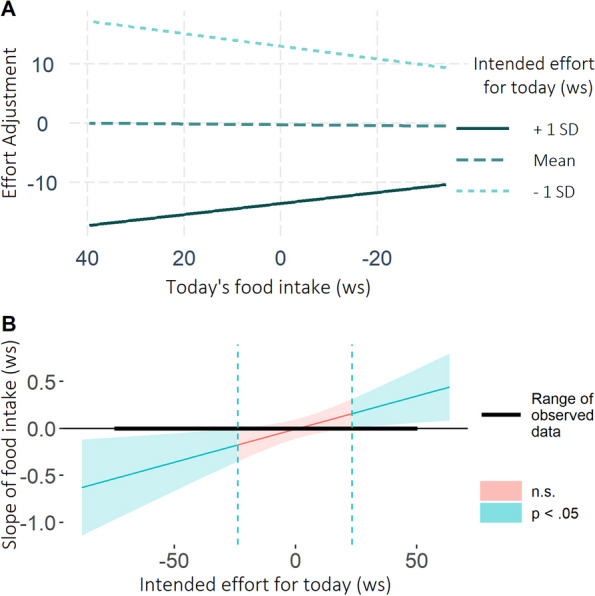
On high-effort days, self-regulatory effort increased more after low (i.e., dietary ‘success’) than high food intake (i.e., dietary ‘failure’). **A** Interaction between *intended effort for today* and *today’s food intake* on *effort adjustements*. **B** Johnson-Neyman-Plot illustrating for which level of intended effort the amount of food intake was significantly related to the self-regulatory effort adjustments

The model testing whether today’s amount of food intake influenced effort adjustments for tomorrow on days participants tried to eat according to their goal (Model 2: implicit monitoring / appraisal) yielded a significant interaction between *Food Intake_today* and *Intended_effort_for_today* (*b* = -0.007, β = -0.05, *t* (22.30) = -2.48, *p* = 0.021). The lower, solid line in Fig. 5A (and significant positive slope in Fig. 5B) indicates that on days for which the intended effort was *high* (+ 1 SD), self-regulatory effort showed a stronger relative *in*crease for low vs. high food intake. Thus, model 1 and 2 both showed that effort increased more strongly after dietary ‘success’ than after dietary ‘failure’. Though unrelated to our hypotheses, the upper, dashed line in Fig. 5A (and significant negative slope in Fig. 5B), show the reverse pattern: On days for which the intended effort was *low* (-1 SD), low food intake was related to a stronger relative *de*crease in dietary effort than high food intake.

### Exploratory analyses: effort Adjustments based on food intake depended on food type

**Fig. 6:**
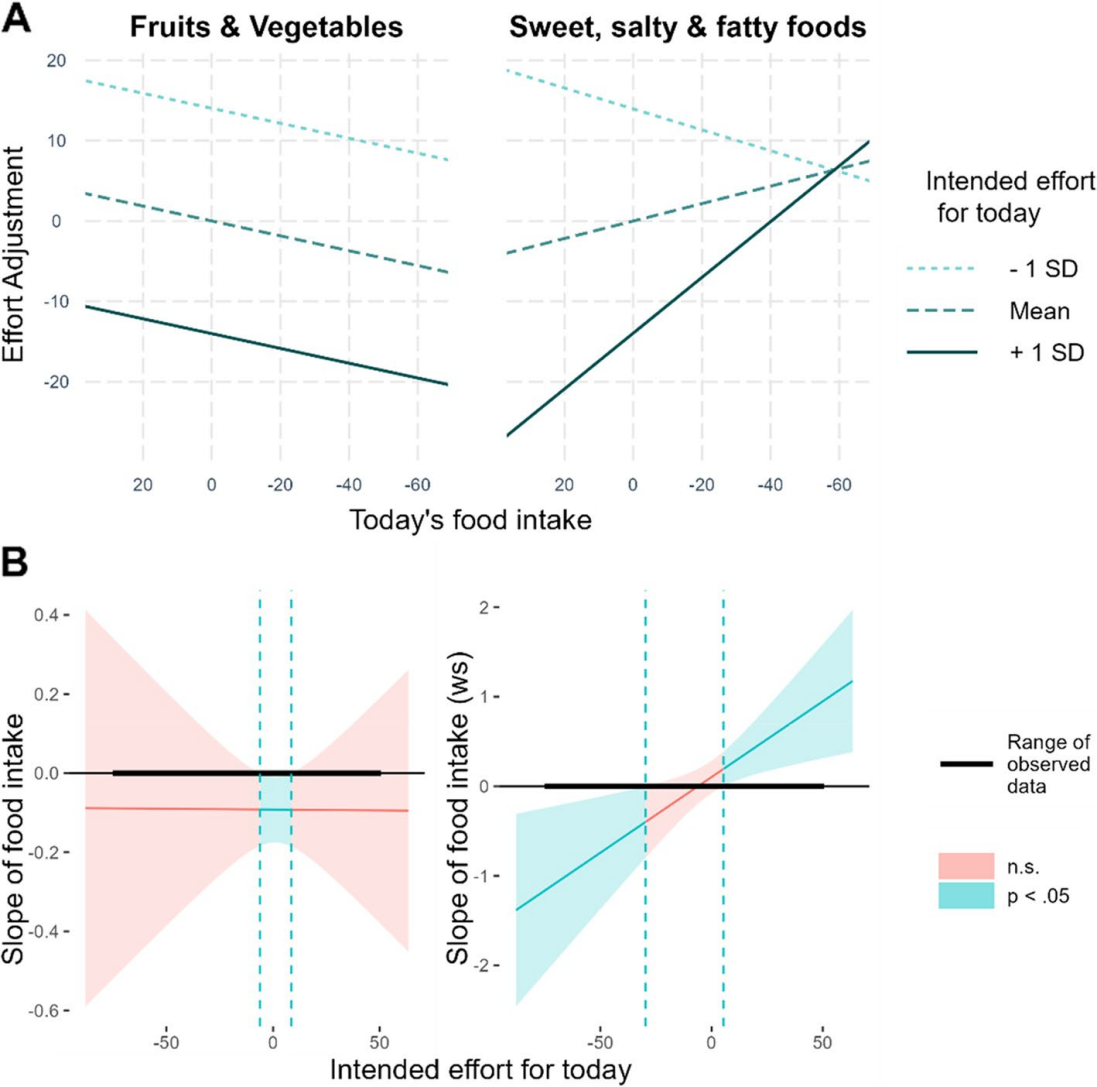
Intake-related effort adjustments for high intake of sweet, salty, and fatty foods, but not for high intake of fruits and vegetables. **A** Interaction between *intended effort for today* and *today’s food intake* for fruits and vegetables (left) as well as for sweet, salty and fatty foods (right); **B** Johnson-Neyman-Plots display for which level of intended effort the amount of food intake was significantly related to the regulatory effort adjustments

As the amount of food intake does not necessarily define dietary ‘success’ or ‘failure’ for people who aim to decrease their intake of some specific foods we explored *food type* as potential moderator. As we expected that most participants aimed to increase intake of fruits and vegetables and decrease intake of sweets, salty snacks and fatty foods, the amount of intake for these food types on a specific day were separately averaged and the interaction with the dummy coded variable *FoodType* (0 = fruits and Vegetables; 1 = sweets, salty snacks and fatty foods) was added as a fixed effect to equation 2^1^. The 3-way interaction between *intended_effort_for_today*, food *intake_today* and *FoodType* was significant (Table 2). Separately analyzing the interaction between *intended_effort_for_today*, and *intake_today* for the different type of foods revealed that the above-described *in*crease in regulatory effort after dietary ‘success’ (compared to ‘failure’) was significant for intake of sweets, salty snacks, and fatty foods (*b* = -0.02, β = -0.05, *t* (705.61) = -3.08, *p* = 0.002) but not for fruits and vegetables (*b* = 0, β = 0, *t* (1284.76) = -0.05, *p* = 0.960). As depicted by the right, solid line of Fig. 6A, on days for which the intended effort was *high* (+ 1SD), regulatory effort increased when participants ate a *low* amount of sweets, salty snacks and fatty foods (i.e., dietary ‘success’) compared to when they ate *high* amounts of these foods (i.e., dietary ‘failure’). The follow-up Johnson-Neyman plot (Fig. 6B) indicates that this relative *in*crease in effort is significant as soon as participants´ intended effort (i.e., goal strength) was higher than their personal average. This pattern was not present for fruits and vegetables as depicted on the left side of Fig. 6A and B. Thus, adjustments in regulatory effort were restricted to prior intake of sweets, salty snacks, and fatty foods.

**Table 2:** Intake-related effort adjustments by food type

	**Effort Adjustment by intake depends on food type**			
*Predictors*	*Estimates*	*std. Beta*	*df*	*p*
Intercept	-1.41	0.00	2437.85	0.338
Day (ws)	0.10	0.02	143.50	0.417
Food intake (ws)	0.09	0.03	94.23	**0.032**
Intended effort (ws)	-0.99	-0.71	254.87	** < 0.001**
Food Type (ws)	-0.01	-0.00	2939.87	0.984
Food intake (bs)	0.00	0.00	2807.51	0.905
Intended effort (bs)	0.01	0.01	2437.70	0.395
Dataset (bs)	-0.46	-0.02	2285.37	0.359
Intake × Effort (ws)	-0.00	-0.00	2494.16	0.989
Intake × Food Type (ws)	-0.20	-0.07	2296.70	0.053
Effort × Food Type (ws)	0.00	0.00	2957.49	0.885
Intake × Effort × Food Type (ws)	-0.02	-0.08	3013.28	**0.014**
N _participants_	175			
Observations	3284			
Marginal R^2^ / Conditional R^2^	0.537 / NA			

‘(ws)’ within-subject effect; ‘(bs)’ between-subject effect

### Exploratory analyses: effort adjustments based on food intake depended on trait-level success in dieting

**Fig. 7:**
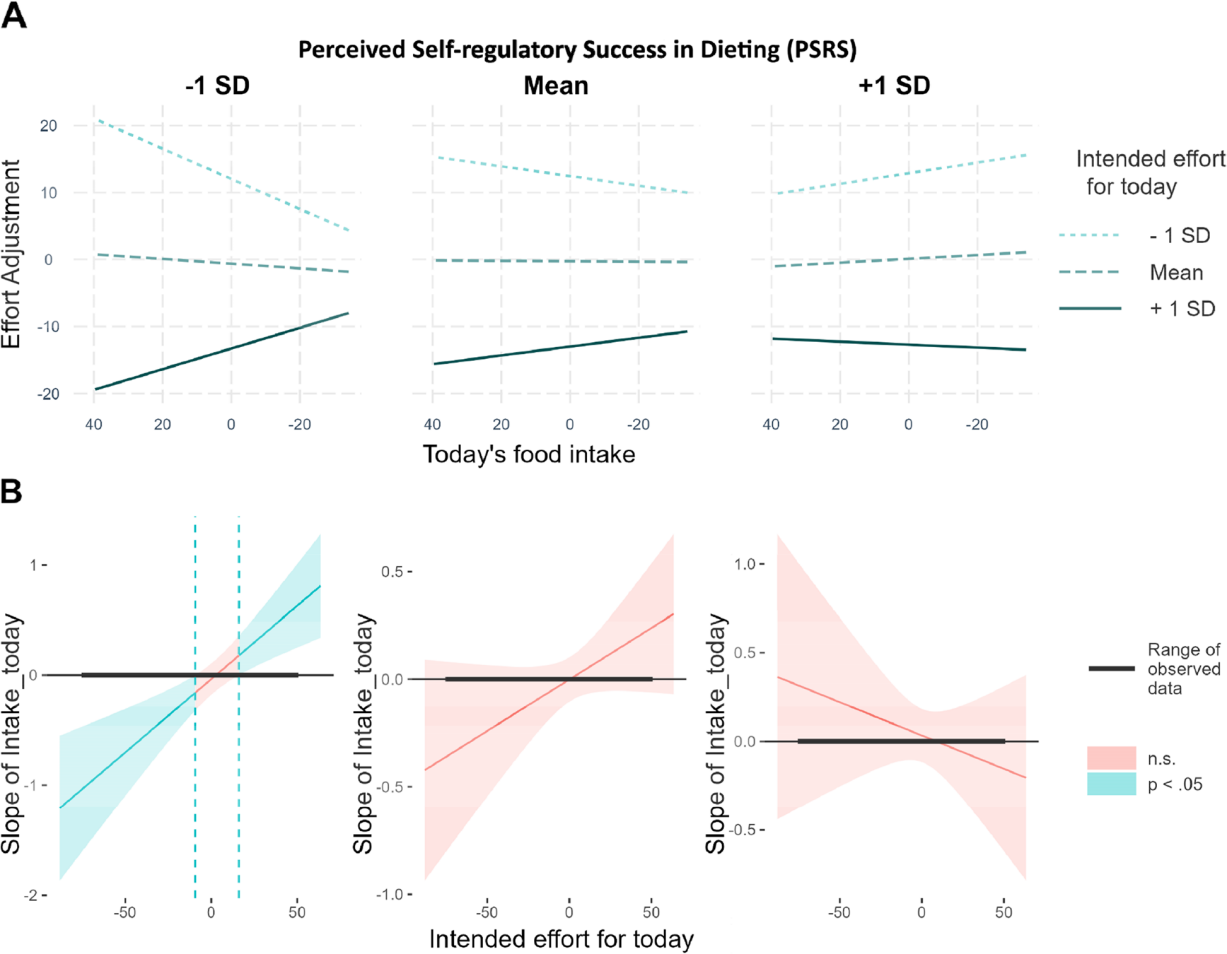
Intake-related effort adjustments in unsuccessful dieters but not in successful dieters. **A** Interaction between *intended effort for today* and *today’s food intake* for self-perceived unsuccessfull (right), moderately successful (middle), and successful dieters (left); **B** Johnson-Neyman-Plots display for which level of intended effort the amount of food intake was significantly related to the regulatory effort adjustments

To further explore whether self-regulatory effort adjustments differed between participants with high vs. low self-regulatory success in dieting we added a 3-way interaction term between the *PSRS-score*, *intended_effort_for_today* and *food intake_today* as a fixed effect to equation 1. The significant 3-way interaction (Table 3) indicates that the degree to which participants adjusted their self-regulatory effort to previous ‘success’ or ‘failure’ depended on their PSRS-score. We found the above-described *in*crease in regulatory effort after dietary ‘success’ (compared to ‘failure’) in individuals with low PSRS scores only (solid line in the left panel of Fig. 7A). Individuals with high PSRS scores did not significantly adjust their self-regulatory effort to former dietary ‘success’ vs. ‘failure’ (solid line in the left panel of Fig. 7A). In the model with goal-related evaluation of food intake, the interaction between goal-congruent eating and the PSRS score was not significant (see Additional file 1: Appendix A.5).

**Table 3:** Intake-related effort adjustments by the perceived self-regulatory success in dieting

	**Effort Adjustment**			
*Predictors*	*Estimates*	*std. Beta*	*df*	*p*
Intercept	1.82	-0.01	1416.34	0.535
Day (ws)	0.11	0.02	130.86	0.227
Food intake (ws)	0.00	0.00	141.35	0.938
Intended effort (ws)	-0.90	-0.67	162.44	** < 0.001**
PSRS (bs)	0.84	0.02	1398.87	0.298
Food intake (bs)	-0.06	-0.02	1407.21	0.207
Intended effort (bs)	0.01	0.01	1424.66	0.698
Dataset (bs)	0.07	0.01	1429.56	0.926
Intake × Effort (ws)	-0.00	-0.03	30.77	0.110
Intake × PSRS (ws/bs)	-0.08	-0.02	145.15	0.527
Effort × PSRS (ws/bs)	-0.01	-0.01	174.79	0.896
Intake × Effort × PSRS (ws/bs)	0.02	0.06	48.76	**0.007**
N _participant_	174			
Observations	1638			
Marginal R^2^ / Conditional R^2^	0.484 / NA			

‘(ws)’ within-subject effect; ‘(bs)’ between-subject effect

## Discussion

We used intensive longitudinal EMA data on dietary behaviors in individuals motivated to lose weight in order to examine how one’s current progress towards dietary goals feeds back to self-regulatory effort, which we regard as a fundamental part of behavior change. Participants *in*creased their self-regulatory effort more strongly after dietary ‘success’ (i.e., eating was congruent with personal goals) than after dietary ‘failure’ (i.e., eating was incongruent with personal goals). These results support the self-reinforcing hypothesis derived from motivational models of self-regulation (H2) and contradict the hypothesis derived from cybernetic models of self-regulation (H1). Exploratory analyses further showed that effort adjustments depended on food type and participants’ trait level perceived self-regulatory success in dieting (i.e., weight management).

Contrary to predictions of cybernetic models of self-regulation, instances of dietary ‘failure’ did not lead to subsequent increases in self-regulatory effort, nor did eating in line with dietary goals lead to decreased self-regulatory effort, so-called motivational “coasting” [40]. These findings are particularly noteworthy, as such calibrating feedback processes are at the core of cybernetic approaches to self-regulation. In the eating context, they are however only supported by one experimental study in which participants allowed themselves to decrease effort after instructed to reflect on past ‘successes’, i.e., behaviors that were in line with their dieting goal (Study 1; [18]). In the current study, by contrast, participants *increased* regulatory effort more after dietary ‘success’ than after dietary ‘failure’. This effect was present both when goal-related eating was operationalized with participants’ evaluations of their behavior as goal-(in)congruent (i.e., explicit progress monitoring) as well as when it was operationalized with the amount of food intake, specifically low amounts of sweets and snacks (i.e., implicit progress monitoring).

Thus, current findings instead support SCT [2], that assumes that meeting dietary goals on a given day – so-called ‘mastery experiences’ – facilitates self-efficacy, i.e., self-evaluative beliefs that one can achieve a healthy diet. Because such positive changes in self-evaluative cognitions after dietary ‘success’ can in turn increase the likelihood of goal progress (i.e., increase in regulatory effort), the reciprocal relationship between goal progress and self-evaluative cognition can initiate an upward spiral of healthy eating. On the downside, failing to meet dietary goals may initiate a downward spiral of unhealthy eating as negative changes in self-evaluative cognitions could turn a single dietary ‘failure’ into a full-blown relapse. That is, dieters may abandon their dietary goal completely and revert to unhealthy intake patterns (abstinence violation effect, [41]). Previous EMA studies found evidence for the presence of negative self-evaluative cognition after dietary ‘failure’. Specifically, participants reported stronger feelings of guilt and failure as well as lower willpower and lower self-efficacy after a dietary lapse (i.e., temptations that were given into) compared to situations unrelated to eating (i.e., random prompts) and/or compared to dietary successes (i.e., temptations that were *not* given into; [22–24]). This dovetails with our finding of generally more positive and less negative affect after dietary ‘success’ compared to ‘failure’ (see Additional file 1: Appendix B).

Importantly, changes in regulatory effort after goal-related eating differed between individuals in the current study. Participants who considered themselves as unsuccessful dieters responded most strongly in line with predictions of motivational theories. That is, they decreased their self-regulatory effort after dietary ‘failure’ relative to dietary ‘success’. One reason for this could be that unsuccessful dieters may be generally less committed to their dietary goals as weak goals and related cognitions (e.g., attitudes, perceived behavioral control, intentions) are assumed to be less stable over time [42–44]. Specifically, weak goals are assumed to be less resistant to external and internal events (e.g., temptations, emotions, changes in daily routines) than strong goals, and thus individuals with weak goals may be also more responsive to self-monitored successes or failures in generally. Indeed, our supplementary analyses showed that unsuccessful dieters generally varied more strongly in their intended regulatory effort compared to successful dieters (Additional file 1: Appendix B). Besides weak goals, different attribution styles could be another reason why unsuccessful dieters show more adjustments in their self-regulatory efforts than successful dieters. Specifically, effort adjustments may be more likely when individuals attribute dietary ‘successes’ and ‘failures’ to themselves (i.e. internally) rather than to external causes. In the case of substance use disorder, such internal attributions lead to stronger changes in self-evaluative cognitions as well as to a complete abandonment of abstinence goal [28]. Indeed, also in the eating context, research has shown that attributing dietary ‘failure’ to internal factors can reduce the time between two binges [45] and may be harmful for healthy eating, as this attribution style is more common in individuals with eating disorders than in those without [46]. Regardless of the underlying reasons *why* unsuccessful dieters respond stronger to their own goal-related eating than successful dieters (i.e., whether it is due to internal attributions or weak goals), a tendency to decrease regulatory effort after instances of dietary ‘failure’ may contribute to an ongoing failure to follow one’s diet, as described in terms of a downward spiral earlier. Such personal experiences of unsuccessful dieting are probably reflected in the PSRS and may thus explain the relationship between effort adjustments and self-reported dietary success. Although not the primary focus of the current study, it is noteworthy that self-regulatory effort increased more strongly after participants ate much (compared to when they ate little) on days with low intentions to self-regulate eating (i.e., ‘cheat days’). These findings – unexplained by cybernetic and motivational theories—are consistent with self-licensing accounts. In those, individuals allow themselves to indulge inasmuch they can justify (or ‘license’) this behavior for themselves [47]. Such *justified* indulgence are assumed to be beneficial in the long run because they threaten perceived self-regulatory abilities less than *unjustified* dietary ‘failures’ [48, 49]. Thus, according to the self-licensing literature, flexibly allowing unhealthy eating on a limited basis may keep people motivated to stick to their dietary goals. This may possibly explain why we found increased self-regulatory effort after days with low intentions to self-regulate eating and high food intake.

### Implications for interventions

The present study is, at least to our knowledge, the first to examine the dynamic interplay of goal-related progress monitoring and regulatory effort adjustments with regards to dietary behaviors. Previous studies had typically focused on single instances of self-control failure or only assessed long-term weight changes (e.g., across 4 weeks) which do not fit the iterative and temporally fine-grained character of self-regulation. For example, evidence for cybernetic models comes mainly from intervention studies. In these studies, self-monitoring is employed to enable the detection of discrepancies between behavioral performance and set goals and has been shown to be a successful intervention part across a variety of health behaviors including weight reduction [16]. Current findings however suggest that prompting self-monitoring through daily intake-related questions, a practice commonly employed in calorie tracking apps, may also inadvertently trigger maladaptive responses to dietary ‘failure’. These responses include decreased self-regulatory effort and, as shown by prior studies, negative self-evaluations [22–24]. Thus, while monitoring the discrepancies between one’s behavioral performance and set dietary goals (i.e., dietary ‘failures’) is probably a crucial prerequisite to initiate behavioral change, the risk of maladaptive responses to dietary ‘failures’ should also be considered when designing dietary interventions. One potential strategy to mitigate maladaptive responses to dietary ‘failures’ involves attributing failures to situation specific external causes, in conjunction with acknowledging that understanding these causes increases chances of future dietary ‘success’ [50]. Yet, future research should confirm whether decreases in regulatory effort transfer to changes in food intake, an important outcome in dietary interventions. Further, in contrast to most intervention studies, the current sample included only few participants with an elevated Body Mass Index. Thus, even though our findings align with previously found changes in negative self-evaluative cognitions in individuals with obesity, future studies should confirm if current results indeed generalize to individuals who typically enroll in dietary interventions.

### Limitations

The continuous but bounded answer scales may have limited interpretation of findings as they hindered participants to report a further increase or decrease in dietary effort when effort was already close to the scales’ boundaries. This probably resulted in the main effects of intended effort: Participants were generally more likely to decrease their efforts after high-effort days than after low-effort days. Thus, we can only interpret *relative* changes in effort. That is, effort can only be claimed to *in*crease after dietary ‘success’ than after dietary ‘failure’ in comparison to how strong effort decreases in general due to intake-independent factors such as bounded scales or regression to the mean.

Results are further limited by the sampling scheme. Neither cybernetic nor motivational theories indicate the time scales at which regulatory efforts would be adjusted, i.e., whether this would be hours, days, or weeks. Our static sampling scheme with one evening measure of intended effort could have therefore overlooked other patterns of faster or slower effort adjustments. Previous EMA studies, for example, sampled cognitive-affective responses to lapses directly after the incident instead of in the evening only [22–24]. Measuring intended effort just before bedtime with no upcoming meals on that day might differ from an approach with more frequent day measurements. Thus, future studies should investigate the influence of different, and potentially flexible and personalized, sampling schemes. Higher sampling frequencies may also help to investigate whether the changes in intended regulatory effort are translated into the target behavior.

## Conclusion

Using two operationalization of goal-(in)congruent dietary behavior, we showed that diet motivated individuals increased their self-regulatory effort more after dietary ‘success’ than after ‘failure’. This points to self-reinforcing feedback loops implied in SCT and other motivational theories but speaks against calibrating feedback loops which are at the core of cybernetic models. The observed relationships with participants’ trait dietary success highlights the importance of these feedback processes as a key mechanism for the future development and evaluation of dietary interventions. Specifically, current results suggest that interventions should consider potential maladaptive responses to dietary ‘failures’ such as decreased self-regulatory effort.

### Supplementary Information

**Additional file 1.**

## Data Availability

Data and analyses scripts are available on OSF: https://osf.io/p2kq5/.

## Abbreviations

EMA: Ecological momentary assessment
SCT: Social-Cognitive Theory
PSRS: Perceived Self-regulatory Success in Dieting Scale
bs: Between-subject
ws: Within-subject

## Footnotes

1 We also added *FoodType* as a random effect per participant but did not model the maximal random effect structure due to convergence issues. Instead, we compared Akaike ‘s Information Criterion for different models and selected the best fitting random effects structure, namely correlated random main effects for intentions_today, food intake_today, FoodType, and day per participant.
