# Supplementary material for: Goal pursuit increases more after dietary success than after dietary failure: examining conflicting theories of self-regulation using ecological momentary assessment
Source: Int J Behav Nutr Phys Act. 2024 Feb 26;21:24. doi: 10.1186/s12966-024-01566-x (PMC10895756; doi:10.1186/s12966-024-01566-x)
Supplement: Supplementary file 1 — Additional file 1. [file 12966_2024_1566_MOESM1_ESM.docx]

Supplemental Materials for ‘Goal pursuit increases more after dietary success than after dietary failure: Examining conflicting theories of Self-regulation using Ecological Momentary Assessment’

Hannah van Alebeek^a^, Christopher M. Jones^b^, Julia Reichenberger^a^, Björn Pannicke^a^, Benjamin Schüz^c^, & Jens Blechert^a^

a. Paris-Lodron-University of Salzburg, Department of Psychology, Centre for Cognitive Neuroscience, Hellbrunner Str. 34, 5020 Salzburg, Austria

b. Medical Faculty Mannheim, Heidelberg University, Germany

c. Institute for Public Health and Nursing Research, University of Bremen, Bremen, Germany

**Appendix A**

**Table A.1**

Dietary effort was significantly adjusted based on participants’ explicit evaluations whether eating was goal-congruent in both datasets.

|  | **Dataset 1: Effort Adjustment** | | | | | **Dataset 2: Effort Adjustment** | | | | |  |
| --- | --- | --- | --- | --- | --- | --- | --- | --- | --- | --- | --- |
| *Predictors* | *Estimates* | *std. Beta* | *df* | *p* |  | *Estimates* | *std. Beta* | *df* | *p* |  |  |
| Intercept | 1.66 | -0.01 | 763.06 | 0.546 |  | -4.84 | -0.00 | 740.78 | 0.097 |  | |
| Day (ws) | -0.14 | -0.03 | 250.24 | 0.253 |  | 0.39 | 0.07 | 69.57 | 0.052 |  | |
| Goal-congruent eating (ws) | 0.11 | 0.10 | 70.67 | **0.004** |  | 0.08 | 0.06 | 119.96 | **0.040** |  | |
| Intended effort (ws) | -0.87 | -0.63 | 88.02 | **<0.001** |  | -1.02 | -0.72 | 83.89 | **<0.001** |  | |
| Goal-congruent eating (bs) | 0.01 | 0.00 | 765.69 | 0.841 |  | 0.02 | 0.01 | 740.31 | 0.667 |  | |
| Intended effort (bs) | -0.01 | -0.01 | 759.10 | 0.671 |  | 0.02 | 0.02 | 736.10 | 0.527 |  | |
| N | 92 _participantss_ | | | | | 83 _participants_ | | | | |  |
| Observations | 821 | | | | | 821 | | | | |  |
| Marginal R^2^ / Conditional R^2^ | 0.459 / NA | | | | | 0.530 / NA | | | | |  |

*Note.* ‘(ws)’ within-subject effect; ‘(bs)’ between-subject effect

**Table A.2**

Dietary effort was significantly adjusted after goal-related food intake in both datasets.

|  | **Dataset 1: Effort Adjustment** | | | | | **Dataset 2: Effort Adjustment** | | | | | | | | |  |  |
| --- | --- | --- | --- | --- | --- | --- | --- | --- | --- | --- | --- | --- | --- | --- | --- | --- |
| *Predictors* | *Estimates* | *std. Beta* | *df* | *p* |  | *Estimates* | | *std. Beta* | | *df* | | *p* | |  |  |  |
| Intercept | 9.10 | -0.01 | 635.32 | **0.025** |  | | -5.16 | | -0.01 | | 714.92 | | 0.243 | | |  |
| Day (ws) | -0.14 | -0.03 | 91.17 | 0.258 |  | | 0.41 | | 0.07 | | 68.21 | | **0.038** | | |  |
| Food intake (ws) | -0.04 | -0.03 | 65.08 | 0.555 |  | | 0.06 | | 0.03 | | 79.28 | | 0.451 | | |  |
| Intended effort (ws) | -0.88 | -0.64 | 88.58 | **<0.001** |  | | -0.98 | | -0.70 | | 74.36 | | **<0.001** | | |  |
| Food intake (bs) | -0.15 | -0.06 | 624.18 | **0.017** |  | | 0.01 | | 0.00 | | 698.03 | | 0.887 | | |  |
| Intended effort (bs) | -0.00 | -0.00 | 655.51 | 0.882 |  | | 0.02 | | 0.02 | | 719.87 | | 0.395 | | |  |
| Effort × Intake | -0.01 | -0.06 | 65.25 | **0.050** |  | | -0.01 | | -0.06 | | 40.53 | | **0.027** | | |  |
| N | 92 _participants_ | | | | | 83 _participants_ | | | | | | | | |  |  |
| Observations | 821 | | | | | 821 | | | | | | | | |  |  |
| Marginal R^2^ / Conditional R^2^ | 0.480 / NA | | | | | 0.538 / NA | | | | | | | | |  |  |

*Note.* ‘(ws)’ within-subject effect; ‘(bs)’ between-subject effect

**Table A.3**

Intake-related adjustments in self-regulatory effort depended on the type of foods in dataset 1 only.

|  | **Dataset 1: Effort Adjustment** | | | | | | **Dataset 2: Effort Adjustment** | | | | | |  |  |  |  |
| --- | --- | --- | --- | --- | --- | --- | --- | --- | --- | --- | --- | --- | --- | --- | --- | --- |
| *Predictors* | *Estimates* | *std. Beta* | *df* | *p* |  | *Estimates* | | | | *std. Beta* | *df* | *p* | |  |  |  |
| Intercept | 0.79 | -0.03 | 1544.48 | 0.684 |  | | | -4.61 | -0.00 | | 1494.81 | **0.041** | | | |  |
| Day (ws) | -0.14 | -0.03 | 69.99 | 0.100 |  | | | 0.43 | 0.07 | | 73.42 | **0.046** | | | |  |
| Food intake (ws) | 0.10 | 0.04 | 1609.48 | 0.070 |  | | | 0.09 | 0.03 | | 48.36 | 0.214 | | | |  |
| Intended effort (ws) | -0.85 | -0.66 | 135.13 | **<0.001** |  | | | -1.05 | -0.75 | | 128.76 | **<0.001** | | | |  |
| Food Type | 0.13 | 0.01 | 1517.74 | 0.887 |  | | | 0.01 | 0.00 | | 1435.71 | 0.987 | | | |  |
| Food intake (bs) | 0.02 | 0.01 | 1545.70 | 0.761 |  | | | 0.00 | 0.00 | | 1479.32 | 0.937 | | | |  |
| Intended effort (bs) | -0.00 | -0.00 | 1593.37 | 0.892 |  | | | 0.02 | 0.02 | | 1500.37 | 0.237 | | | |  |
| Intake × Effort (ws) | 0.01 | 0.05 | 1561.27 | **0.023** |  | | | -0.01 | -0.03 | | 1387.03 | 0.145 | | | |  |
| Intake × Food Type (ws) | -0.02 | -0.03 | 1607.48 | 0.853 |  | | | -0.33 | -0.10 | | 1183.53 | **0.037** | | | |  |
| Effort × Food Type (ws) | -0.00 | -0.00 | 1480.24 | 0.962 |  | | | 0.00 | 0.00 | | 1481.44 | 0.942 | | | |  |
| Intake × Effort × Food Type (ws) | -0.04 | -0.17 | 1585.11 | **<0.001** |  | | | -0.00 | -0.02 | | 1508.72 | 0.632 | | | |  |
| N | 92 _participants_ | | | | | | 83 _participants_ | | | | | |  |  |  |  |
| Observations | 1642 | | | | | | 1642 | | | | | |  |  |  |  |
| Marginal R^2^ / Conditional R^2^ | 0.446 / NA | | | | | | 0.571 / NA | | | | | |  |  |  |  |

*Note.* ‘(ws)’ within-subject effect; ‘(bs)’ between-subject effect

**Table A.4**

Effort adjustments based in food intake depended on the perceived success in dieting in dataset 2 only.

|  | **Dataset 1: Effort Adjustment** | | | | | **Dataset 2: Effort Adjustment** | | | | |
| --- | --- | --- | --- | --- | --- | --- | --- | --- | --- | --- |
| *Predictors* | *Estimates* | *std. Beta* | *df* | *p* |  | *Estimates* | *std. Beta* | *df* | *p* |  |
| Intercept | 9.36 | -0.01 | 698.28 | **0.026** |  | -4.14 | -0.01 | 714.93 | 0.352 |  |
| Day (ws) | -0.11 | -0.02 | 71.32 | 0.347 |  | 0.35 | 0.06 | 66.34 | **0.019** |  |
| Food intake (ws) | -0.05 | -0.02 | 63.12 | 0.543 |  | 0.07 | 0.02 | 76.97 | 0.397 |  |
| Intended effort (ws) | -0.87 | -0.63 | 88.81 | **<0.001** |  | -0.91 | -0.69 | 80.99 | **<0.001** |  |
| PSRS (bs) | 0.04 | 0.00 | 683.52 | 0.968 |  | 1.35 | 0.02 | 706.63 | 0.330 |  |
| Food intake (bs) | -0.15 | -0.06 | 698.25 | **0.018** |  | 0.00 | 0.00 | 723.30 | 0.970 |  |
| Intended effort (bs) | -0.01 | -0.00 | 692.27 | 0.827 |  | 0.02 | 0.02 | 712.60 | 0.499 |  |
| Intake × Effort (ws) | -0.00 | -0.03 | 785.49 | 0.276 |  | -0.01 | -0.03 | 14.87 | 0.167 |  |
| Intake × PSRS (ws/bs) | -0.03 | -0.01 | 62.79 | 0.856 |  | -0.21 | -0.04 | 89.50 | 0.289 |  |
| Effort × PSRS (ws/bs) | -0.05 | -0.02 | 94.72 | 0.692 |  | -0.00 | -0.01 | 88.31 | 0.994 |  |
| Intake × Effort × PSRS (ws/bs) | 0.01 | 0.04 | 761.46 | 0.140 |  | 0.03 | 0.07 | 20.72 | **0.025** |  |
| N | 91 _participants_ | | | | | 83 _participants_ | | | | |
| Observations | 817 | | | | | 821 | | | | |
| Marginal R^2^ / Conditional R^2^ | 0.470 / NA | | | | | 0.500 / NA | | | | |

*Note.* ‘(ws)’ within-subject effect; ‘(bs)’ between-subject effect

**Table A.5**

Adjustments in dietary effort based on participants’ goal-congruent eating did not significantly differ in individuals with either higher or low self-regulatory success in dieting.

|  | **Combined Datasets: Effort Adjustment** | | | | |  |
| --- | --- | --- | --- | --- | --- | --- |
| *Predictors* |  | *Estimates* | *std. Beta* | *df* | *p* |  |
| Intercept |  | -1.11 | 0.00 | 1437.00 | 0.568 |  |
| Day (ws) |  | 0.10 | 0.02 | 131.37 | 0.295 |  |
| Intended effort (ws) |  | 0.10 | 0.08 | 136.02 | **<0.001** |  |
| Goal-congruent eating (ws) |  | 0.67 | 0.01 | 1409.52 | 0.432 |  |
| PSRS (bs) |  | -0.93 | -0.69 | 161.41 | **<0.001** |  |
| Intended effort (bs) |  | 0.00 | 1440.01 | 0.952 | 0.968 |  |
| Goal-congruent eating (bs) |  | 0.01 | 0.01 | 1432.83 | 0.746 |  |
| PSRS × Goal-congruent (ws/bs) |  | -0.03 | -0.01 | 119.10 | 0.654 |  |
| Dataset (bs) |  | -0.30 | -0.01 | 1450.07 | 0.679 |  |
| N | 174 _participants_ | | | | |  |
| Observations | 1638 | | | | |  |
| Marginal R^2^ / Conditional R^2^ | 0.473 / NA | | | | |  |

*Note.* ‘(ws)’ within-subject effect; ‘(bs)’ between-subject effect

**Appendix B**

**B.1. Participants’ explicit goal-congruent monitoring changes positive and negative affect**

As cybernetic and motivational approaches assume that changes in affect accompany or signal the need for adjustments in self-regulatory effort, we additionally tested whether past dietary ‘success’ and ‘failure’ lead to changes in positive and negative affect using equation 3 and 4. In both datasets, participants rated on each EMA-signal how happy, enthusiastic, relaxed, calm, active, irritated, worried, depressed, bored, nervous or stressed from 0 (‘not at all’) to 100 (‘very’). The former, and latter five emotion ratings were averaged to achieve a positive and negative affect score, respectively. These variables were lagged by one EMA signal and then subtracted from the current signal to calculate the dependent variable, that is, the change in positive and negative affect from one EMA signal to the next.

For the model which defined dietary ‘success’ and ‘failure’ with participants’ explicitly monitored goal-congruent eating (equation 3), a significant main effect of *Goal-congruent eating* on the *changed positive affect* indicated that positive affect *in*creased the more participants rated their food intake in the in-between time window as goal-congruent (*b* = .02, β = .04, *t* (139.72) = 3.09, *p* = .002). Opposite effects were found for the model predicting *changed negative affect* (Figure B.1). Here, a significant main effect of *Goal-congruent eating* indicated that negative affect increased with more goal-*in*congruent ratings (*b* = -.01, β = -.03, *t* (114.58) = -2.78, *p* = .006).

For the model which defined dietary ‘success’ and ‘failure’ with participants’ implicit goal-related monitoring, namely with the amount of food intake on days they intended to eat healthily (equation 4), there was neither a significant effect of the interaction on the change in positive effect nor on the changed negative affect.

| (3) | *Changed Positive/Negative Affect ~ Goal-congruent eating (ws) + Prior affect (ws) + Goal-congruent eating (bs) + Prior affect (bs) + Day + Dataset + (Goal-congruent eating (ws) + Prior affect (ws) + Day \| Subject)* |
| --- | --- |
| (4) | *Changed Positive/Negative Affect ~ Food Intake (ws) × Intended_effort_for_today (ws) + Prior affect (ws) + Goal-congruent eating (bs) + Prior affect (bs) + Day + Dataset + (Food Intake (ws) × Intended_effort_for_today (ws) + Prior affect (ws)+ Day \| Subject)* |

**Figure B.1**

Relative to participants’ goal-*in*congruent eating, explicit ratings that food intake was goal-congruent eating increased positive affect while it decreased negative affect.**
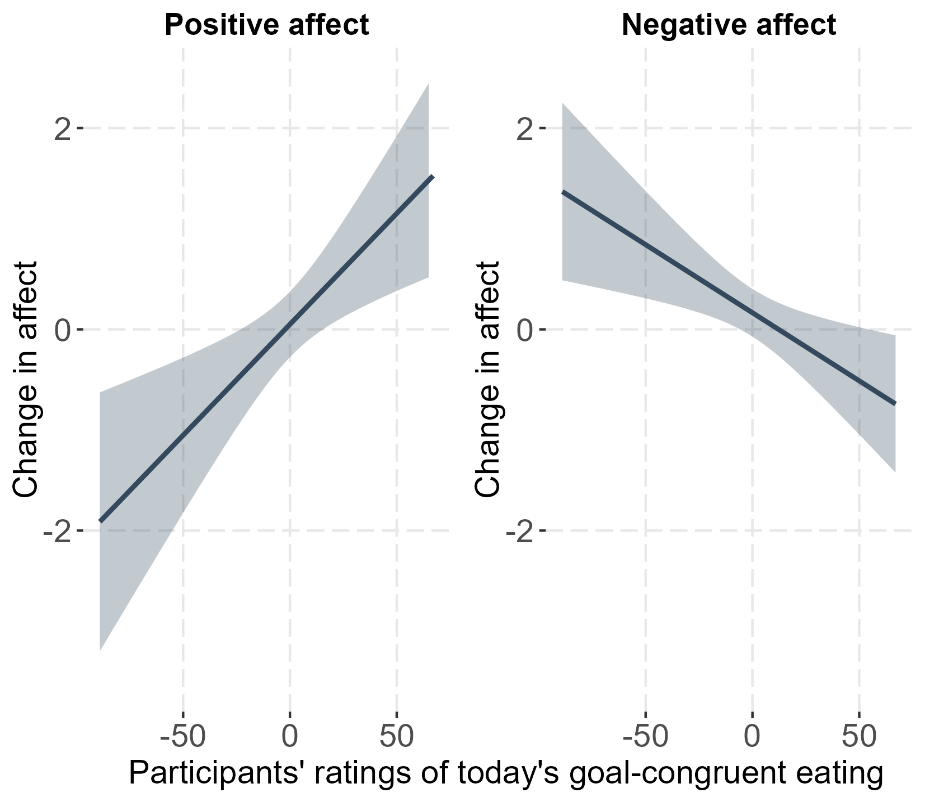
**

**B.2. Are the effects sensitive to different cutoffs for participant exclusion?**

To examine whether the significance-levels of our main analyses (Section 3.1) depended on the compliance on participants, we re-analyzed the models using different cutoffs for compliance and plotted the p-values as seen in Figure B.4. When dietary ‘success’ and ‘failure’ was operationalized with participants’ explicit goal-congruent monitoring the main effect for *goal-congruent eating_today* was strong enough to remain significant across different cutoffs for participant exclusion (Figure B.4A) whereas excluding all participants with compliance lower than 75% (reducing the sample size to 131) led to the disappearance of the significant interaction between *intended_effort_for_today* and *food intake_today* in the model on implicit goal-congruent monitoring (Figure B.4B).

**Figure B.2**


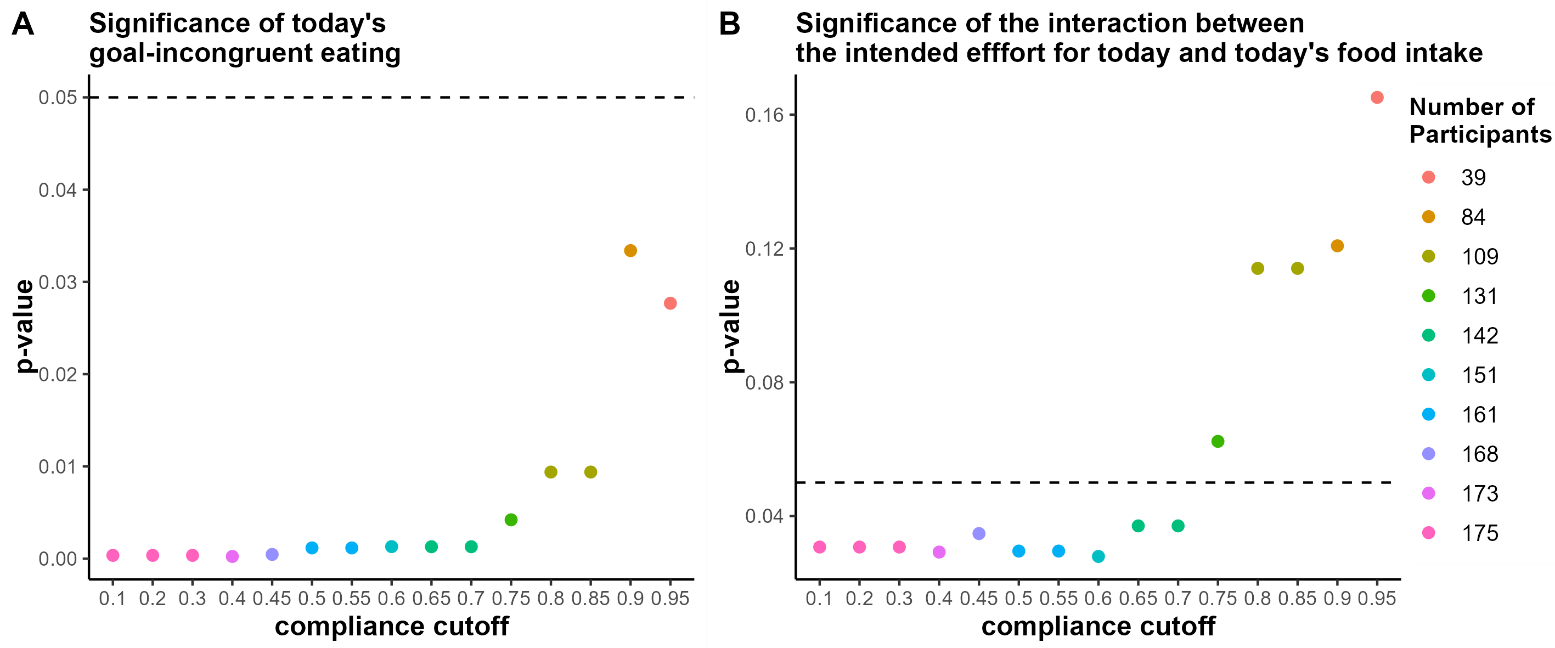


**B.3. Is the trait-level perceived success in dieting related to a greater variability in intended self-regulatory effort?**

To test if unsuccessful dieters show greater temporal variation in their intended self-regulatory efforts than successful dieters, we correlated the scores of the PSRS with first, the standard deviation (SD) of intended effort across the 14 days (i.e., overall variation) and second, the mean square successive difference (MSSD) of intended effort across the 14 days (i.e., changes from one day to the next). As measures for temporal variability were not normally distributed, we used spearman’s rho. Participants with lower scores on the PSRS (i.e., unsuccessful dieters) varied stronger in their intended effort across both operationalization of temporal variability than participants with higher scores on the PSRS (i.e., successful dieters) (SD: *r*(173) = -.22, *p* = .003; MSSD: *r*(173) = -.23, *p* = .002).
